# Dr. E. M. Grace

**Published:** 1911-06

**Authors:** 


### DR. E. M. GRACE.

It is with great regret we have to record the death of Dr. Edward Mills Grace, which took place on May 20th at Thornbury, after some months of failing health.

He was born at Downend on November 28th, 1841, the third son of Henry Mills Grace, a very hard-working country doctor, whose leisure moments in the summer were given up to cricket. The other sons were Henry, Alfred, now at Chipping Sodbury, W. G. and Fred. Henry and G. F., or Fred as everyone knew him, are dead. They were all cricketers, E. M., W. G. and G. F. coming under the public ken more than the others. They were all in the medical profession, and it is not too much to say that their name has been known to more people in the world than any of their medical *confrères* in the West of England. Coached by their father and their uncle, Alfred Pocock, in the orchard adjoining the Chestnuts, their home at Downend, with two or three dogs as fielders, by patient practice at the game, beginning early in their lives and early in the seasons, they built up for themselves a great reputation, and really did more to popularise the national game than any others.

It is not within the scope of this notice to speak of the cricket records and feats which the eldest of the trio, Dr. E. M., had to his name, but it is impossible not to refer to some of them. He began early, and we find that he played against an Eleven of All England in 1855 in a field at the back of the Full Moon Hotel, Bristol, when three of the All England Eleven played in top hats. From 1855 to 1910 he played every year, and probably made more runs in all matches and took more wickets than any other man has ever attained to or is ever likely to attain to. Up to the end of 1903 it has been calculated that his average was 1446 runs per season and 207 wickets. He went to Australia as one of George Parr's team in 1864, and was one of the foremost cricketers in first class matches, playing for Gloucestershire, England, and Gentlemen for the next twenty years. He was Secretary to the Gloucestershire County Club for forty years.

As regards his public life other than cricket, he qualified in 1865, and first practised at Marshfield. He came to Thornbury in October, 1867, and was Surgeon to the Workhouse, Parish Medical Officer and Public Vaccinator from 1867 to 1907. He was also Registrar of Births and Deaths, and Coroner for East Gloucestershire from 1875. He held many clubs—Post Office, Midland Railway, Rational Society, Oddfellows and others. He was Chairman of the first School Board, Chairman of the Parish Council and of the Parochial Sanitary Committee, and also the last Mayor of Thornbury, which office was abolished on the passing of the County Councils Bill. He took a great interest in politics, and was recently Chairman of the Thornbury District of the Thornbury Division of the Conservative Association, and also Chairman of the Tariff Reform League. He was a member of the Berkeley Hunt, and administrator of the Poultry Fund for many years.

He was a man of force and of striking personality. In whatever company he was he was always to the front, and having a boundless confidence in himself he invariably managed to get and to hold his own. Generous, hospitable and bubbling over with a cheery good nature, full of anecdote, with a large fund of adventures to tell the details of, he was a most interesting companion. Entering so largely into the public life of the county where he was so well known, and always having his say, he sometimes said and did things that many did not agree with; but he never nursed a grievance, and was as good friends with his opponents on the next occasion as if no cloud had ever passed between them. He was never afraid, not even of the Press, and in any altercation his pluck and his determination generally brought him the best of the argument. This characteristic pluck was innate, and was evident in his fearless riding to hounds even so lately as when he could not get into the saddle without help, and also evident in his unique fielding close in at " point."

He was a remarkable man, and it will be many years before his words and deeds are forgotten.

He was married four times—in 1868, 1885, 1902 and 1907. There were thirteen children by the first wife, of whom six survive, and five by the second, of whom three survive.

### Local Medical Notes.

